# Laparoscopic-endoscopic cooperative surgery for primary gastric synovial sarcoma: a rare case report and literature review

**DOI:** 10.3389/fonc.2026.1854543

**Published:** 2026-06-02

**Authors:** Ting Xu, Xue Meng, Xuan Gou, Xinyuan Wang, Shuai Luo, Deyuan Zhang

**Affiliations:** 1 Department of Pathology, Zhejiang Provincial People’s Hospital Bijie Hospital (The First People’s Hospital of Bijie), Bijie, Guizhou, China; 2 Department of Pathology, Affiliated Hospital of Zunyi Medical University, Zunyi, Guizhou, China

**Keywords:** gastric body, molecular testing, monophasic, pathological diagnosis, synovial sarcoma

## Abstract

**Background:**

Synovial sarcoma is a malignant mesenchymal tumor, accounting for approximately 5–10% of all soft tissue sarcomas. To date, only 52 cases of primary gastric synovial sarcoma have been reported in the English-language literature. We report a rare case of primary gastric synovial sarcoma in a young woman. To the best of our knowledge, this is the second case in the past decade treated using combined laparoscopic and endoscopic surgery, and the first such case reported in China using this approach.

**Case demonstration:**

A 37-year-old woman was admitted with a 9-month history of abdominal pain and a 3-month history of a gastric body tumor. Initial biopsy at an outside hospital suggested a spindle cell tumor. Immunohistochemistry and molecular testing confirmed gastric monophasic synovial sarcoma. The patient was transferred to our hospital for surgical treatment and underwent regular postoperative follow-up. After nine postoperative months, endoscopic examination revealed no local recurrence. No regional lymph node involvement or distant metastases were identified. The patient remained asymptomatic with good performance status and no gastrointestinal symptoms.

**Conclusions:**

Laparoscopic-endoscopic cooperative surgery is a feasible and effective minimally invasive approach for achieving complete (R0) resection of gastric synovial sarcoma while avoiding open surgery and its associated morbidity.

## Background

Synovial sarcoma (SS) is a mesenchymal tumor characterized by a distinctive histological pattern, most commonly arising near limb joints. However, gastric SS is extremely rare. This report describes a case of gastric SS treated with combined laparoscopic and endoscopic surgery (CELS), and reviews 52 reported cases, including the present case.

## Case presentation

**Figure 1:**
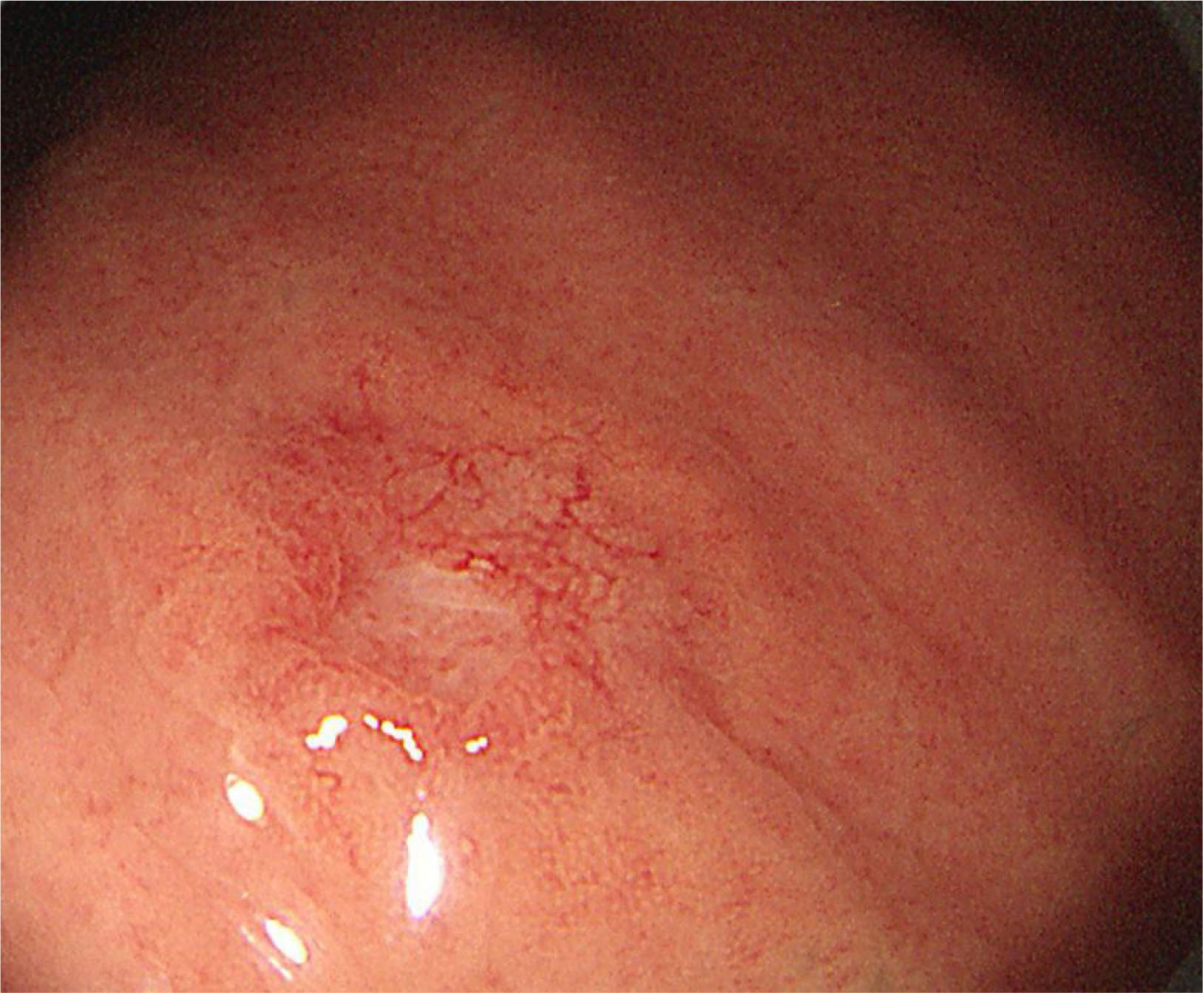
In the upper gastric body along the greater curvature, an O-IIa+IIc lesion measuring approximately 0.8 cm was observed.

**Figure 2:**
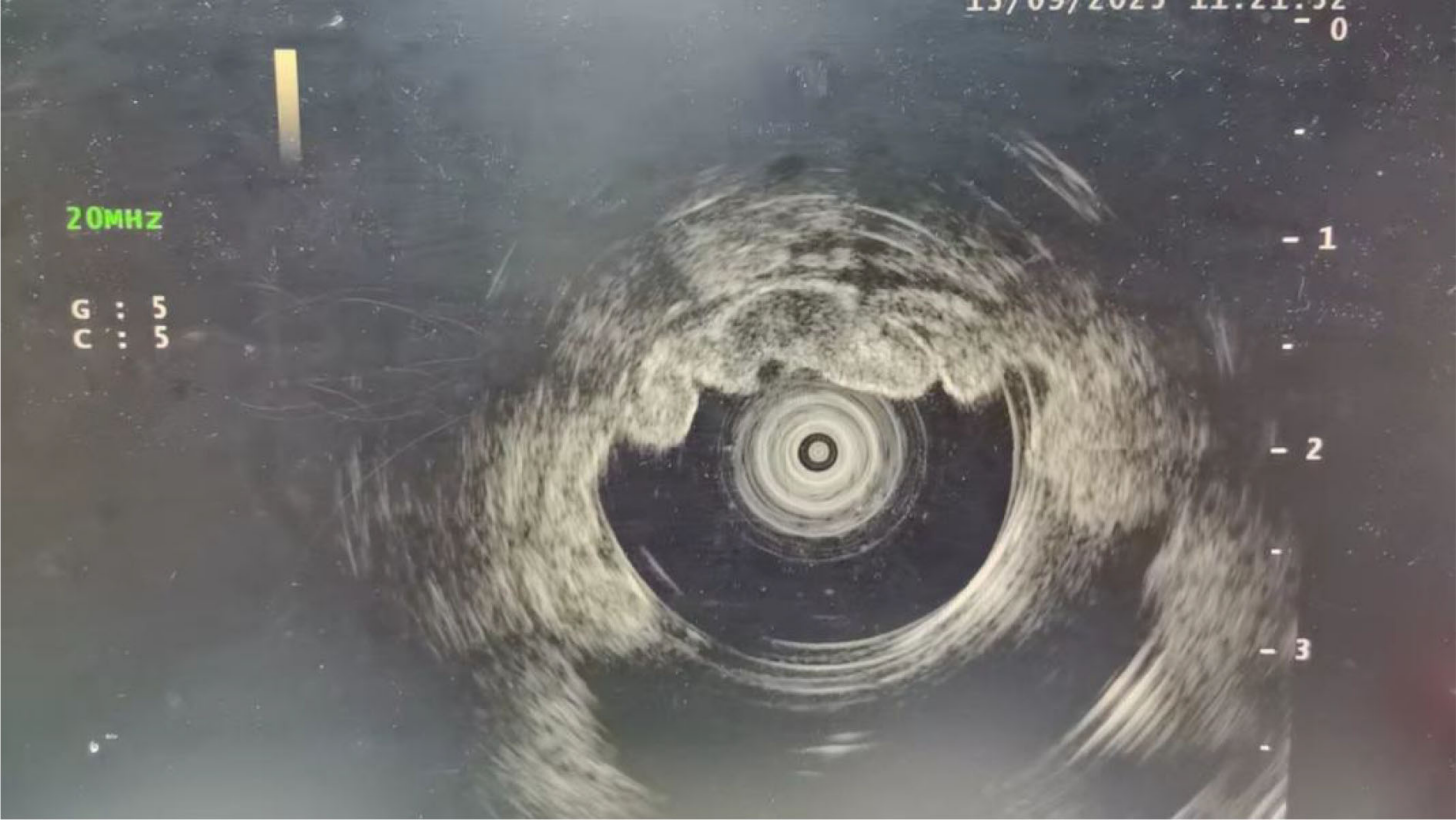
Endoscopic ultrasound showed thickening of the mucosal layer, with clear demarcation between the tumor and the muscularis propria.

A 37-year-old woman presented with a 9-month history of abdominal pain and a 3-month history of a gastric body tumor. The patient had no family history of cancer or genetic syndromes, and an Eastern Cooperative Oncology Group (ECOG) performance status of 0. Nine months earlier, she developed upper abdominal pain without obvious triggers, described mainly as a dull epigastric pain. Three months earlier, a painless esophagogastroduodenoscopy at an outside hospital revealed gastric body changes, suggestive of a tumor. Biopsy indicated a spindle cell tumor, and immunohistochemistry and molecular testing confirmed gastric monophasic SS. The patient was subsequently referred to our hospital for treatment. Standard endoscopy revealed a type 0-IIa+IIc lesion measuring approximately 0.6 cm in the upper gastric body along the greater curvature (**Figure 1**). Endoscopic ultrasonography showed mucosal thickening with preserved wall stratification, and the tumor was clearly separated from the muscularis propria (**Figure 2**).

Preoperative staging included contrast-enhanced chest and abdominal computed tomography (CT), which showed no pulmonary, hepatic, or other metastatic lesions, and no regional lymphadenopathy. The patient initially underwent endoscopic submucosal dissection (ESD) for the gastric tumor. Iatrogenic perforation occurred during submucosal incision, prompting immediate conversion to laparoscopic surgery. After laparoscopic exploration localized the perforation, laparoscopic-assisted local wedge resection was performed to completely remove the full-thickness gastric wall, including the perforation site with an adequate safety margin. The gastric wall defect was then closed by laparoscopic single-layer interrupted suturing (or: linear cutting closure device), and the perforation was repaired simultaneously.

Postoperative recovery was uneventful, and the hospital stay was 6 days. No postoperative complications, including bleeding, anastomotic leak, or stricture, were observed. The patient was discharged in good condition.

**Figure 3:**
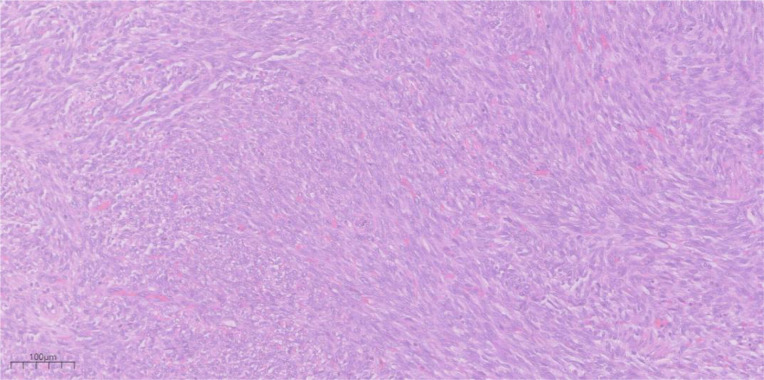
Under low magnification, tumor cells are spindle-shaped and arranged in fascicles (HE ×100).

**Figure 4:**
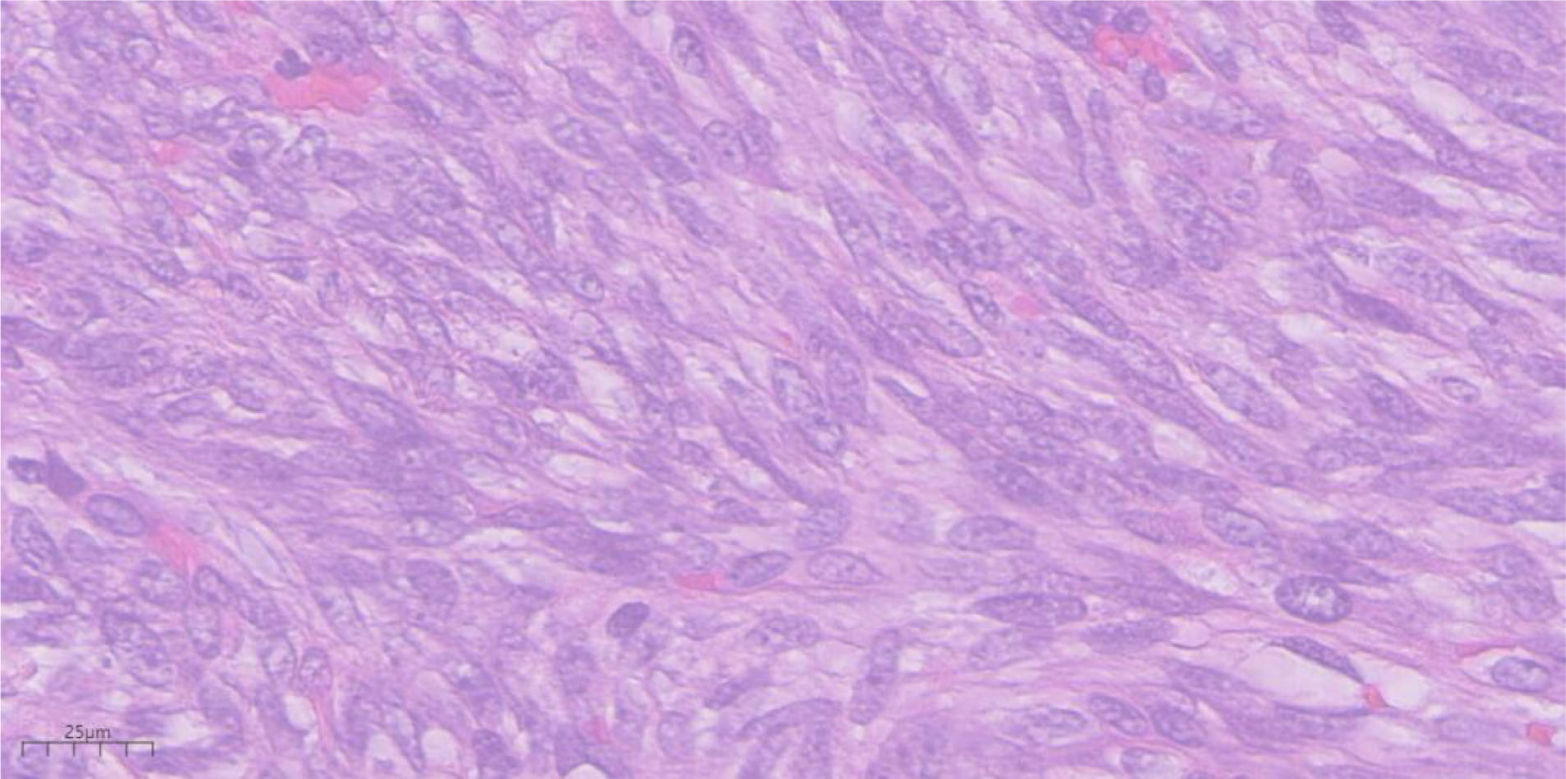
Under high magnification, tumor cells are short spindle-shaped or oval, with scant cytoplasm, slightly basophilic, inconspicuous nucleoli, and no mitotic figures observed (HE ×400).

**Figure 5:**
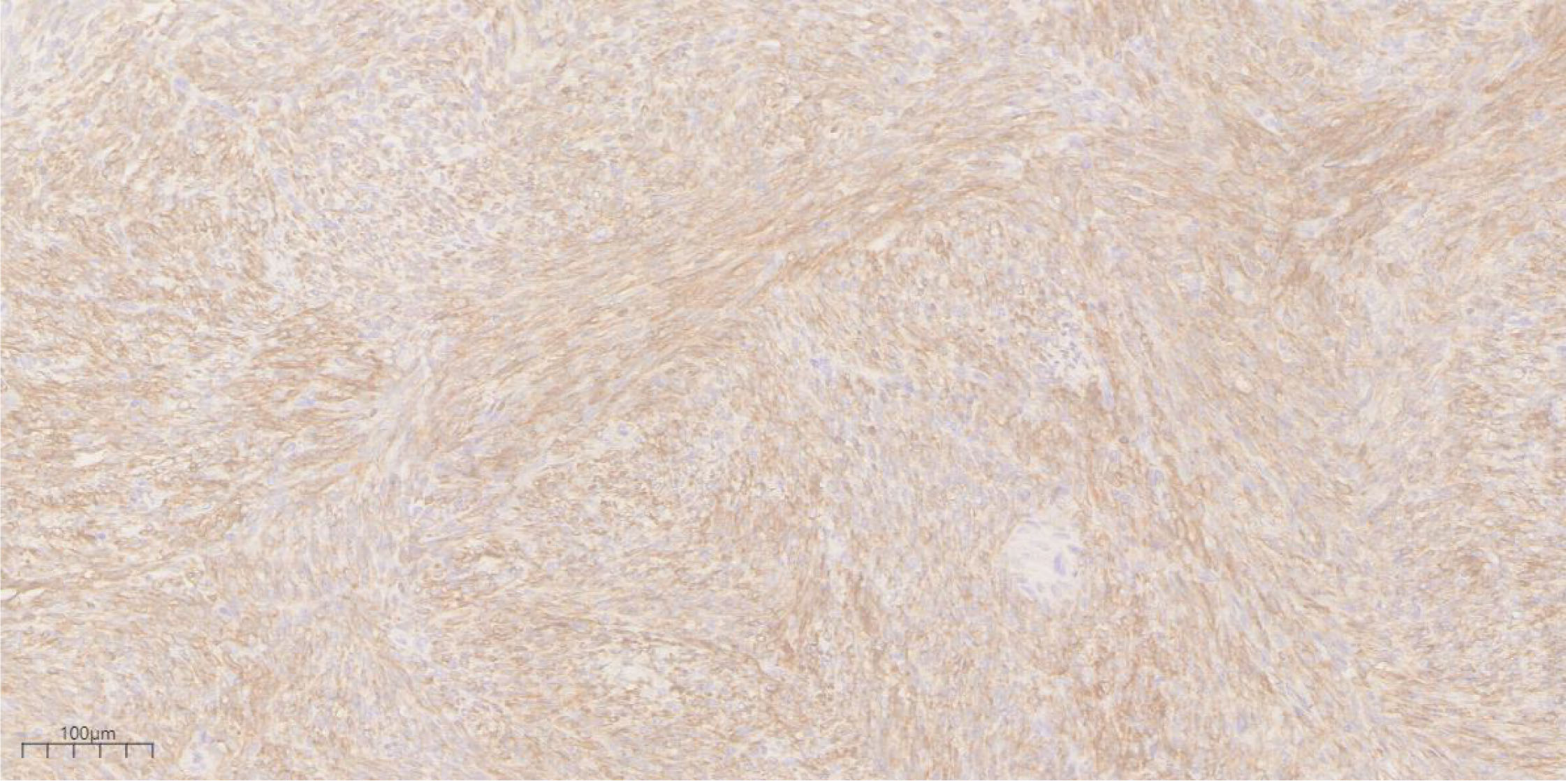
Immunohistochemistry showing BCL-2 positivity (100×).

**Figure 6:**
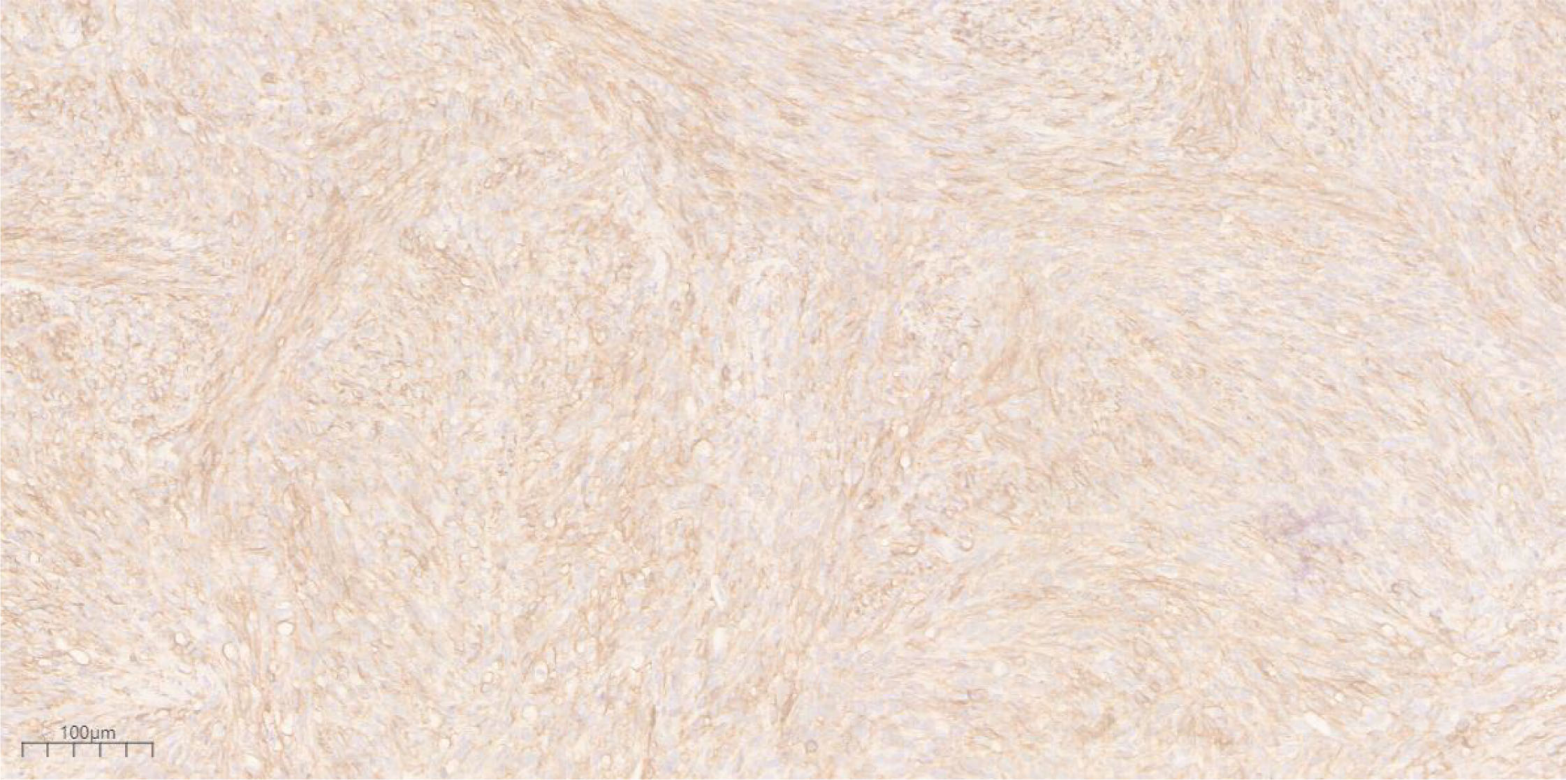
Immunohistochemistry showing CD99 positivity (100×).

Grossly, the specimen consisted of gray-brown gastric mucosal tissue, measuring 5.5 × 4.5 × 0.4 cm, with the nearest and farthest resection margins measuring 1.2 cm and 1.8 cm, respectively. A raised lesion with an ulcerated surface, measuring 0.6 × 0.5 × 0.4 cm, was identified. The cut surface was gray-white and firm. Microscopic examination revealed spindle-shaped cells arranged in fascicles, with spindle or oval-shaped nuclei (**Figure 3**). The cytoplasm was scant, and chromatin was uniform and hyperchromatic, with inconspicuous nucleoli (**Figure 4**). Immunohistochemistry showed CKpan (-), BCL-2 (+) (**Figure 5**), CD99 (+) (**Figure 6**), CD117 (-), DOG1 (-), CD34 (-), STAT6 (-), Desmin (-), SMA (-), S-100 (-), SOX10 (-), and Ki-67 (+20%). Based on microscopic morphology, immunohistochemistry, and molecular testing performed at the outside hospital, a diagnosis of monophasic SS was confirmed. The basal and surgical margins were free of tumor involvement. At the most recent 9-month follow-up, the patient showed no evidence of recurrence or metastasis.

## Discussion

Synovial sarcoma (SS) is a malignant mesenchymal tumor, accounting for <10% of all soft tissue sarcomas. It typically occurs in adolescents and young adults, with a mean age at diagnosis of 39 years, and affects men and women equally (1). A characteristic chromosomal translocation generates the *SYT::SSX* fusion transcript, which genetically characterizes SS; detection of this translocation supports the diagnosis (2). Based on histological pattern and differentiation, SS is classified into three subtypes: monophasic fibrous, composed of spindle cells without epithelial components; biphasic, comprising epithelial and spindle cell components; and poorly differentiated, composed of dedifferentiated spindle and/or round cells (3). SS typically harbors the chromosomal translocations forming *SS18::SSX1*, *SS18::SSX2*, or *SS18::SSX4* fusion genes (4).

Primary gastric SS is an extremely rare mesenchymal tumor, with only limited cases documented. We performed a systematic literature review using the keyword ‘gastric synovial sarcoma’ in PubMed (last search: October 2025). Inclusion criteria were pathologically confirmed primary gastric SS with available molecular diagnostic data. After excluding metastatic cases, 51 previously reported cases were identified.

To our knowledge, only 52 cases of primary gastric SS, including the present case, have been reported in the English-language literature, with clinicopathological features summarized in **Table 1**. The male-to-female ratio is 1:1, age ranges from 13 to 72 years, and the median age at onset is 45 years. The gastric body is the most common tumor site, with an average tumor size of 5.34 cm. Most patients present with ulcers, and the majority of cases are of the monophasic subtype.

**Table 1:** Clinical pathological features and prognostic analysis of 52 cases of primary gastric synovial sarcoma.

Case	Sex	Age	Size (cm)	Subtype	Gastric involvement	Type of surgery and adjuvant treatment	Outcome
1 (2)	M	32	3.5	Monophasic	Below the cardia	Partial gastrectomy	ANED, 8mo
2 (5)	F	61	0.6	Monophasic	Gastric body	Laparoscopic wedge resection of the stomach	ANED, 4mo
3 (6)	M	47	5.2	Biphasic	Gastroesophageal junction	Total gastrectomy and partial esophagectomy	ANED 21 mo
4 (6)	F	55	16	Biphasic	Gastric antrum	Gastrectomy	DOD, 6mo
5 (7)	F	67	0.8	Monophasic fibrous	Body-antrum junction	Partial gastrectomy	ANED 12 mo
6 (7)	M	49	2	Monophasic fibrous, with a poorly differentiated	Gastric body	Segmental/wedge resection	DOD, omental metastases, 29 mo
7 (7)	F	68	2	Monophasic fibrous	Gastric body	Wedge resection of the stomach	ANED, 22 mo
8 (7)	M	29	2.8	Monophasic fibrous	Gastric body	Partial gastrectomy Antrectomy/gastroduodenal resection	ANED, 224 mo
9 (7)	F	54	3	Monophasic fibrous	Antrum, Gastroduodenal junction	Antrectomy/ gastroduodenal resection	Recent case
10 (7)	F	58	3	Monophasic fibrous	Lesser curvature/body	Wedge resection of the stomach	ANED, 21 mo
11 (7)	F	37	4	Monophasic fibrous	Fundus of stomach	Gastrectomy/partial	DOC 48 mo
12 (7)	M	50	6	Monophasic fibrous	Distal fundus	Resection, chemotherapy	ANED, 6 mo
13 (7)	M	42	8	Biphasic	Greater curvature/body	Partial gastrectomy, chemotherapy	DOD, 25 mo
14 (7)	F	66	15	Monophasic fibrous	Fundus of stomach	Gastrectomy/partial esophagectomy	Lost to follow-up
15 (8)	M	42	3	Monophasic fibrous	Lesser curvature	Laparoscopic Subtotal Gastrectomy	ANED, 12 mo
16 (9)	M	54	1.6	Monophasic fibrous	Lesser curvature	Laparoscopic wedge resection of the gastric lesion	NR
17 (6)	F	55	16	Biphasic Morphology	Distal stomach	Partial gastrectomy,	DOD, 6 mo
18 (10)	M	45	0.8	Monophasic fibrous	The anterior wall of the antrum	laparoscopic endoscopic cooperative surgery	ANED, 5mo
19 (11)	F	44	4.7	Monophasic fibrous	Proximal stomach	Laparoscopic wedge resection	ANED, 24mo
20 (12)	F	38	7.5	Monophasic fibrous	Gastric body	Subtotal gastrectomy, chemotherapy	Transfer, 3mo
21 (13)	F	42	3.5	Monophasic fibrous	Gastric body	Partial gastrectomy	ANED, 72mo
22 (14)	M	22	1.7	Monophasic fibrous	Gastric body	Partial gastrectomy	NR
23 (15)	M	62	3.8	Monophasic	Fundus of stomach	Subtotal gastrectomy, chemotherapy	ANED, 9mo
24 (16)	F	50	8	Monophasic	Gastric body	NR	Lost
25 (16)	M	36	6	poorly differentiated	Cardias	NR	36mo,Transfer to Liver
26 (16)	M	37	2	Monophasic	Gastric	NR	Recent
287 (16)	M	26	NR	Monophasic	Gastric	NR	185,Transferto Liver and Lung
28 (16)	M	58	10	Monophasic	Gastric	NR	DOD,6mo
29 (16)	M	21	10	Monophasic	Gastric	NR	Lost,48
30 (16)	M	36	6	Biphasic	Gastric	NR	Lost,12
31 (16)	F	54	3.8	Monophasic	Gastric	NR	Recent
32 (17)	F	49	35	Monophasic	Gastric	Surgery	ANED, 12mo
33 (17)	F	35	12	Monophasic	Gastric	Gastric surgery, chemotherapy	24mo,Transfer to Liver
34 (18)	F	55	1.7	Monophasic	Gastric body	Distal gastrectomy, chemotherapy	ANED, 2mo
35 (19)	F	27	2	Monophasic	Fundus of stomach	Laparoscopic partial gastrectomy	ANED, 6mo
36 (20)	F	57	1.8	Monophasic	Gastroesophageal connection	laparoscopic wedge resection	NR
37 (21)	M	58	6.3	Monophasic	Gastric body	Robotic-assisted laparoscopic surgery and chemotherapy	ANED, 6mo,Transfer to Liver
38 (22)	F	59	2.6	Monophasic	Gastric fundus	Partial gastrectomy	ANED, 60mo
39 (23)	M	13	11	Monophasic	Gastric body and fundus	Total gastrectomy	ANED, 6mo
40 (24)	F	48	9	Monophasic	Pyloric region	Distal gastrectomy and chemotherapy	NR
41 (25)	M	22	1	Monophasic	Gastric body	Laparoscopic partial gastrectomy	ANED, 6mo
42 (25)	F	38	1	Monophasic	Proximal gastric	Submucosal lesion surgical resection	NR
43 (25)	M	72	1.3	Monophasic	Gastric body	Gastric tumor surgical resection	NR
44 (26)	F	43	1	Monophasic	Gastric body	Laparoscopic partial gastrectomy	NR
45 (10)	F	59	NR	Monophasic	NR	NR	NR
46 (10)	M	45	0.8	Monophasic	Gastric body	Total gastrectomy	ANED, 5mo
47 (18)	F	51	1.7	Monophasic	Gastric body	Distal gastrectomy	ANED, 2mo
48 (27)	F	32	3	Monophasic	Gastric body	Partial gastrectomy	ANED, 16mo
49 (28)	F	57	8	Monophasic	Gastric antrum	Billroth II	ANED, 12mo
50 (29)	M	36	9	Monophasic	Gastric antrum	Patient underwent radical subtotal gastrectomy	ANED, 12mo
51 (30)	M	50	1.2	Monophasic	Antrum to lesser curvature	Laparoscopic-endoscopic cooperative surgery	ANED, 6mo
Our case	F	37	0.8	Monophasic	Gastric body	ESD+Laparoscopic lesion resection	Recent cases

ANED, alive with no evidence of disease; DOC, died of other causes; DOD, died of disease, NR, Not Reported; ESD, endoscopic submucosal dissection.

Gastric pain and anemia are the most common clinical manifestations of gastric SS, but are non-specific for diagnosis. Pathology combined with fluorescence *in situ* hybridization (FISH) and reverse transcription polymerase chain reaction (RT-PCR) is considered the gold standard for diagnosing gastric SS. In this case, microscopic examination showed short spindle or oval tumor cells arranged in fascicles with relatively uniform morphology. The cytoplasm was scant, chromatin was uniform and hyperchromatic, nucleoli were inconspicuous, and no mitotic figures were observed. Immunohistochemistry demonstrated positive BCL-2 and CD99 expression. Combined with FISH testing from the outside hospital, positive *SS18* signals (>15%) and *SS18* gene rearrangement were detected, confirming gastric monophasic SS of the gastric body. Surgical margins were free of tumor involvement.

Differential diagnosis included gastrointestinal stromal tumor (GIST), leiomyosarcoma, solitary fibrous tumor, and extraosseous Ewing sarcoma. (1) GIST diagnosis was excluded by negative CD117 and DOG1 staining. (2) Negative SMA, Desmin, and Caldesmon ruled out leiomyosarcoma. (3) Negative STAT6 and CD34 excluded the diagnosis of solitary fibrous tumor. (4) Extraosseous Ewing sarcoma was excluded by weak/patchy CD99 expression, negative NKX2.2, and negative EWSR1 rearrangement (FISH/NGS).

Preoperative contrast-enhanced CT of the chest and abdomen revealed no evidence of distant metastasis, supporting localized disease at presentation.

Among 52 reported gastric SS cases, excluding eight without available records, all others underwent surgical resection using various approaches, including partial, distal, or total gastrectomy, or local tumor resection. Of the 52 cases, seven received combined chemotherapy (7, 9, 15, 21, 24). Two cases, including the present case, were treated with CELS (10). Bulut et al. reported this approach for advanced colonic adenomas and early colorectal cancer (31). For smaller gastric SS tumors, such as the present 0.6-cm lesion, CELS may avoid formal gastric resection, shorten operative time, reduce blood loss, minimize serosal resection, and reduce hospitalization. For early-stage small tumors without local or systemic progression, CELS may represent a safe alternative to standard resection (32). In this case, negative surgical margins allowed a smaller resection area, potentially minimizing postoperative gastric deformity.

In the present case, a 0.4-cm gastric perforation occurred during ESD, necessitating conversion to laparoscopic surgery. The perforation was successfully managed by laparoscopic closure during the subsequent CELS procedure.

Regarding hospitalization, the perforation likely contributed to the extended 6-day hospital stay, as the patient required close observation for possible peritonitis or delayed complications. Fortunately, no intra-abdominal infection, abscess, or fistula developed postoperatively.

Importantly, the perforation did not affect the oncologic outcome. En bloc R0 resection was achieved, with no evidence of tumor spillage or dissemination. The patient remains recurrence-free at 9 months of follow-up, although longer surveillance is needed. This case highlights that while ESD is a valuable diagnostic and therapeutic tool for early gastric lesions, perforation is a recognized risk that may require surgical conversion. However, with prompt recognition and appropriate laparoscopic management, oncologic outcomes need not be compromised.

Given the limited evidence in current literature, the roles of adjuvant chemotherapy, radiotherapy, and intraoperative lymph node dissection remain unclear. For small lesions, CELS may be an effective approach, as it can achieve negative margins, which is the gold standard for treatment.

## Conclusion

Because of the rarity of gastric SS, misdiagnosis is likely in clinical practice, and an accurate diagnosis requires combined pathological examination and molecular testing. CELS offers a safe and effective minimally invasive option for localized gastric SS, enabling complete tumor resection while avoiding open surgery. For this rare tumor, this surgical approach warrants further investigation; however, its long-term efficacy still requires validation through the accumulation of additional cases and extended follow-up.

## Data Availability

The original contributions presented in the study are included in the article/Supplementary Material. Further inquiries can be directed to the corresponding author.
